# Computational modeling of the effects of amyloid-beta on release probability at hippocampal synapses

**DOI:** 10.3389/fncom.2013.00001

**Published:** 2013-01-25

**Authors:** Armando Romani, Cristina Marchetti, Daniela Bianchi, Xavier Leinekugel, Panayiota Poirazi, Michele Migliore, Hélène Marie

**Affiliations:** ^1^Laboratory of Molecular Mechanisms of Synaptic Plasticity, European Brain Research InstituteRome, Italy; ^2^Department of Physics, University La SapienzaRome, Italy; ^3^Institut des Maladies Neurodégénératives, UMR 5293, CNRS and Université de BordeauxBordeaux, France; ^4^Institute of Molecular Biology and Biotechnology, Foundation of Research and Technology-HellasHeraklion, Crete, Greece; ^5^Institute of Biophysics, National Research CouncilPalermo, Italy

**Keywords:** amyloid-beta, hippocampus, computational modeling, release probability, neuronal output

## Abstract

The role of amyloid beta (Aβ) in brain function and in the pathogenesis of Alzheimer's disease (AD) remains elusive. Recent publications reported that an increase in Aβ concentration perturbs pre-synaptic release in hippocampal neurons. In particular, it was shown *in vitro* that Aβ is an endogenous regulator of synaptic transmission at the CA3-CA1 synapse, enhancing its release probability. How this synaptic modulator influences neuronal output during physiological stimulation patterns, such as those elicited *in vivo*, is still unknown. Using a realistic model of hippocampal CA1 pyramidal neurons, we first implemented this Aβ-induced enhancement of release probability and validated the model by reproducing the experimental findings. We then demonstrated that this synaptic modification can significantly alter synaptic integration properties in a wide range of physiologically relevant input frequencies (from 5 to 200 Hz). Finally, we used natural input patterns, obtained from CA3 pyramidal neurons *in vivo* during free exploration of rats in an open field, to investigate the effects of enhanced Aβ on synaptic release under physiological conditions. The model shows that the CA1 neuronal response to these natural patterns is altered in the increased-Aβ condition, especially for frequencies in the theta and gamma ranges. These results suggest that the perturbation of release probability induced by increased Aβ can significantly alter the spike probability of CA1 pyramidal neurons and thus contribute to abnormal hippocampal function during AD.

## Introduction

Alzheimer's disease (AD) is a progressive neurodegenerative brain disorder that primarily affects memory-encoding brain areas such as the hippocampus. Despite the intense research efforts provided to understand this pathology, the primary cause of the disease is still not clear and different hypotheses have been proposed (de la Torre, 2011). The amyloid hypothesis states that one of the first events in AD is an altered processing of the amyloid precursor protein (APP) leading to accumulation of the Amyloid beta (Aβ), especially in the hippocampus and related brain areas. It is postulated that this increase in Aβ leads to abnormal neuronal function and disruption of brain information processing, which in turns leads to the cognitive deficits observed in AD patients.

Among the many effects of Aβ accumulation [reviewed in (Walsh and Teplow, 2012)], its action on glutamatergic synaptic function has been intensively studied over the last decades, but its exact role still remains elusive. There is now evidence that Aβ acts as a positive endogenous modulator of glutamate release (Abramov et al., 2009; Parodi et al., 2010). In particular, Abramov et al. demonstrated that an acute enhancement of endogenous Aβ leads to an increase in the initial release probability (*p*_0_) at the CA3-CA1 synapses of the hippocampus, without altering postsynaptic function or intrinsic neuronal excitability (Abramov et al., 2009). This increase in *p*_0_ was associated with an increase in vesicle depletion, an Aβ-induced phenomenon also observed in another recent study (Parodi et al., 2010). Whether this acute effect of Aβ is physiological or pathological remains to be elucidated. Importantly, how this enhancement in *p*_0_ influences synaptic short-term plasticity of the synapse and the firing probability of the CA1 output neuron has not been investigated.

To address this issue, we performed a computational study using a realistic CA1 pyramidal neuron model (Bianchi et al., 2012), complemented with a widely used model of short-term plasticity (Tsodyks et al., 1998) adapted to reproduce the Aβ-induced enhancement of *p*_0_. Our results suggest that this synaptic modification significantly alters synaptic integration over a wide range of input frequencies and that, when stimulated with natural input patterns obtained from *in vivo* recordings, a neuron in the increased-Aβ condition exhibits significant changes in its firing probability and in its response to theta and gamma frequencies. The model thus predicts that the observed Aβ-induced alterations in release probability within the hippocampus can significantly alter the information flow and contribute to the pathogenesis of AD.

## Materials and methods

### Computational model

All simulations were performed using the NEURON simulation environment (v7.2) (Hines and Carnevale, 1997), with a previously published model of hippocampal CA1 pyramidal neurons (Bianchi et al., 2012). To implement synaptic inputs suitable to model changes in release probability and synaptic integration, for each synapse we used the kinetic scheme introduced by Tsodyks et al. (1998) and widely used to study synaptic transmission mechanisms and short-term plasticity effects (Tsodyks et al., 1998).

Briefly, in this model a synapse contains a finite amount of ‘resources’, which can be divided in three fractions: recovered (*x*), active (*y*), and inactive (*z*), in a dynamical relation to each other. At the arrival of a spike (at time *t*_sp_), a fraction *p* of recovered resource is activated, quickly inactivated with a time constant τ_in_ and then recovered with a time constant τ_rec_ according to the following equations:

$$\begin{array}{l} \frac{dx}{dt}=\frac{z}{\tau_{\text{rec}}}-px\delta (t-t_{\text{sp}})\\ \frac{dy}{dt}=-\frac{y}{\tau_{\text{in}}}+px\delta (t-t_{\text{sp}})\\ \frac{dz}{dt}=\frac{y}{\tau_{\text{in}}}-\frac{z}{\tau_{\text{rec}}} \end{array}$$

with *x* + *y* + *z* = 1 and *y*_0_ = *z*_0_ = 0, *x*_0_ = 1.

*p* indicates the effective use of the synaptic resources of the synapses and can be seen as the average release probability of a quantal model. During repeated stimulation *p* can increase due to facilitation. In particular, at the arrival of a spike (*t* = *t*^*i*^_sp_) *p* is incremented by a factor *U*(1 − *p*^−^), where *p*^−^ is the last pre-spike value of *p*, so that the post-spike value is *p*^+^ = *p*^−^ + *U*(1 − *p*^−^). After the spike, instead, *p* decays to baseline with a time constant τ_facil_, (Tsodyks et al., 1998), so that in the interval between the arrival of two spikes, *t* ∈ (*t*^*i*^_sp_, *t*^*j*^_sp_):

$$\frac{dp}{dt}=-\frac{p}{\tau_{\text{facil}}}$$

*U* determines the increase in the value of *p* with each spike and coincides with the value of *p* reached upon the arrival of the first spike, i.e., *p*_0_ = *p*(*t*^1^_sp_) = *U*. It is worth noting that *p* is incremented before *x* is converted to *y*.

The resulting excitatory post-synaptic current (EPSC) is proportional to the active resource:

EPSC(t)=Ay(t)

where *A* is the absolute synaptic strength, corresponding to the maximum EPSC obtained by activating all the resources.

The values used for all parameters are discussed in the Results section. Since the findings in Abramov et al. (2009) are principally mediated by AMPA receptors, we did not explicitly include NMDA receptors in the CA1 neuron model.

To model the activation of a group of afferent fibers from CA3 pyramidal neurons through the Schaffer collaterals, ten synapses were distributed randomly on the apical trunk between 100 and 400 μm from the soma. Each synapse in the model represents the effect of a population of synapses activated in the oblique dendrites. A range of peak synaptic conductances (weights) was explored. All simulations were repeated 10 times redistributing synaptic location and weights. Results are expressed as mean ± SEM (standard error of the mean, SEM).

### *In vivo* recordings

The action potentials (APs) discharged by individual CA3 pyramidal neurons during open-field exploration of a large square plywood box (120 × 120 cm, 50 cm high) were recorded (1000 × amplification, 1–9000 Hz bandpass, digitized with 16 bit resolution, 20 kHz sampling rate using DataMax system, RC-electronics, Santa Barbara, CA) from male Long-Evans rats (300–500 g) implanted with eight adjustable-tetrodes (Szabo et al., 2001), as described in a previous study (Hirase et al., 2001). Repeated exploration of the box was facilitated by randomly dispersing chocolate pieces on the floor. Water was freely available. A LED attached to the headstage was used to track the position of the animal. Localization of electrodes was histologically confirmed to be the CA3 pyramidal layer. Spike sorting was performed by using the Neuroscope—ndmanager—klusters software package (Hazan et al., 2006). For stimulation of the model neuron, a 10 min period of these recordings was used.

### Data analysis and statistics

**Figure 1:**
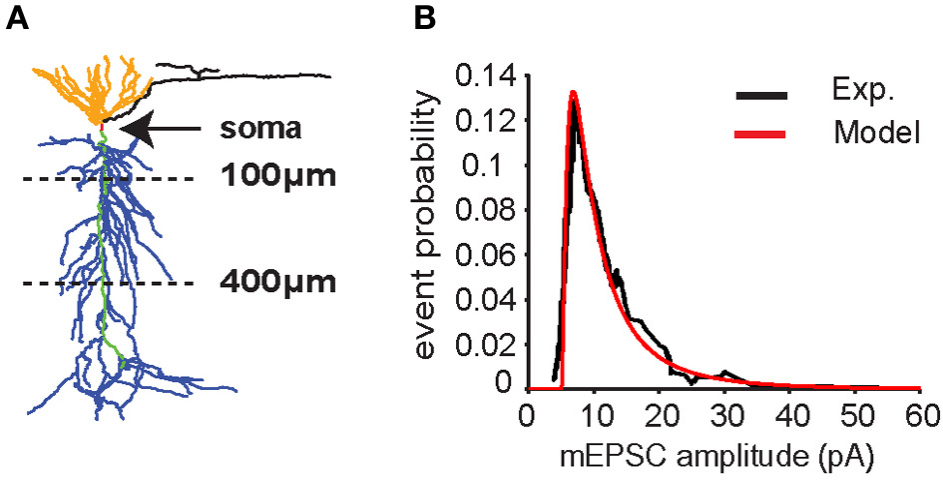
**CA1 pyramidal neuron model. (A)** Neuron morphology. Synapses were placed on the apical trunk (green) between 100 and 400 μm from the soma (red) to mimic inputs coming from CA3 pyramidal neurons to apical dendrites (blue) through the Schaffer's collaterals. **(B)** Distribution of miniature excitatory postsynaptic current (mEPSC) amplitudes in somatic recording (soma clamped at −70 mV) according to experimental data (Ito and Schuman, 2009) (black line). Data were fitted with a lognormal distribution (μ = 1.6, σ = 1, *x*_0_ = 5) (red line).

Curve fitting (Figures 1B and 5B) was performed using Matlab (v. 7.8.0.347).

**Figure 2:**
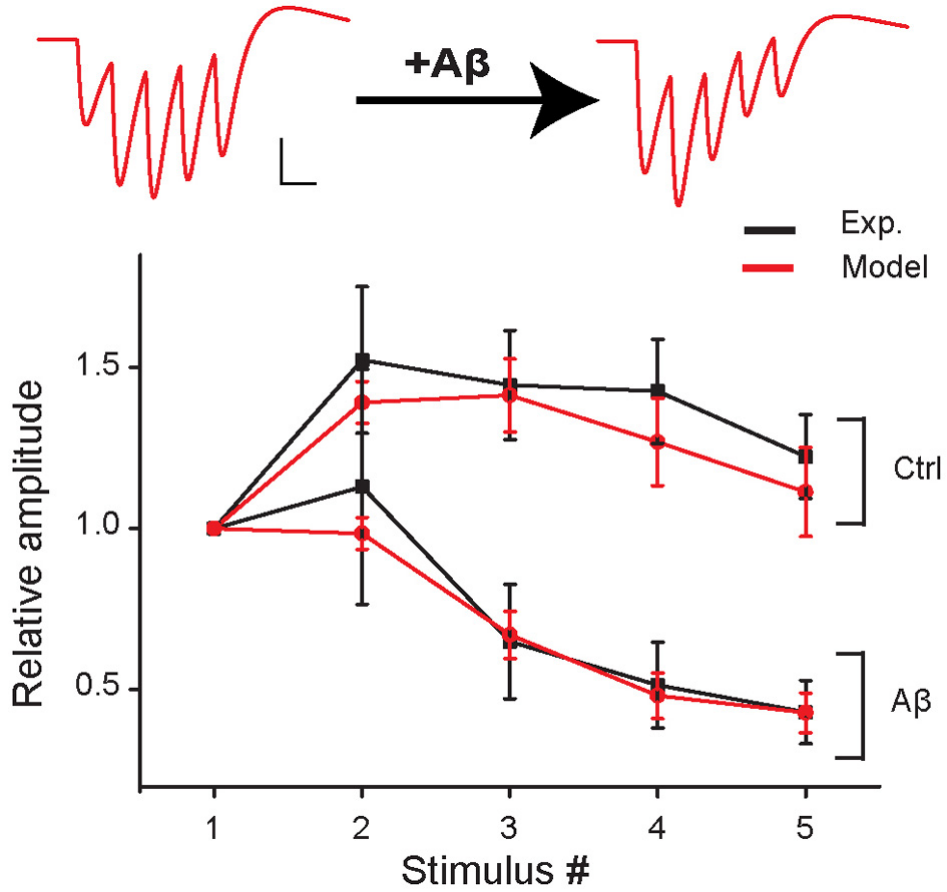
**Short-term plasticity during 5 stimuli at 100 Hz in control and in the increased-Aβ condition.** Average relative somatic EPSC amplitude (normalized to first response) against stimulus number in experimental data (Abramov et al., 2009) (black curve, soma clamped at −70 mV) and in the model (red curve). An example of traces produced with the model is shown on top. Scale bars: 1 pA, 10 ms.

The parameters in Tsodyks' model (Figure 2) were obtained using the least square error (LSE) method. The values found were the ones which minimize the sum of squares of the differences between experimental and model points.

Data points (Figures 5C–E) were normally distributed (Shapiro–Wilk test), which allowed us to use a paired *t*-test for statistical analysis. The average probability distributions (Figures 5D–F) were compared using the Kolmogorov–Smirnov test.

Results were considered significantly different if *p* < 0.05.

## Results

### Validation of the model

The neuron model used for all simulations is represented in Figure 1A. All the simulations are performed with 10 synapses, with each synapse assumed to model several real synaptic inputs (*n* = 15), as described in the paragraph “Computational model.” The peak synaptic conductances used in each simulation were drawn from a lognormal distribution fitting for miniature EPSC (mEPSC) recorded in the CA1 neuron apical trunk as reported in Ito and Schuman (2009) (Figure 1B).

We first found a set of values for the synaptic parameters giving good qualitative agreement with the experimental findings of Abramov et al. (2009) under control conditions. The initial release probability (*p*_0_) was set to 0.15 according to the median of the release probability distribution found by Abramov et al. (2009). The peak synaptic conductances were adjusted to qualitatively reproduce the first EPSP peak measured by Abramov et al. (2009) (*model*: 17.6 ± 3.9 pA, *experiment*: 17.4 ± 6.7 pA). This corresponds to about 150 individual real active synapses. The other model parameters were set to replicate the experimental AMPAR-mediated EPSCs evoked in paired recordings of excitatory neurons by five presynaptic APs at 100 Hz (presynaptic neuron, current clamp; postsynaptic neuron, voltage clamp −70 mV, Abramov et al., 2009). The best fit was obtained with τ_in_ = 1 ms, τ_rec_ = 50 ms, and τ_facil_ = 200 ms (see Figure 2, control).

We next mimicked the Aβ-dependent increase in pre-synaptic release probability and found that an increase of *p*_0_ from 0.15 to 0.36 was sufficient to reproduce the Aβ-induced change in EPSC dynamics observed in response to a train of pre-synaptic APs by Abramov et al. (Figure 2).

These results validated the model against a specific set of experimental data, allowing us to predict the possible functional consequences under more physiological input patterns.

### Additional hypotheses on Aβ effects

**Figure 3:**
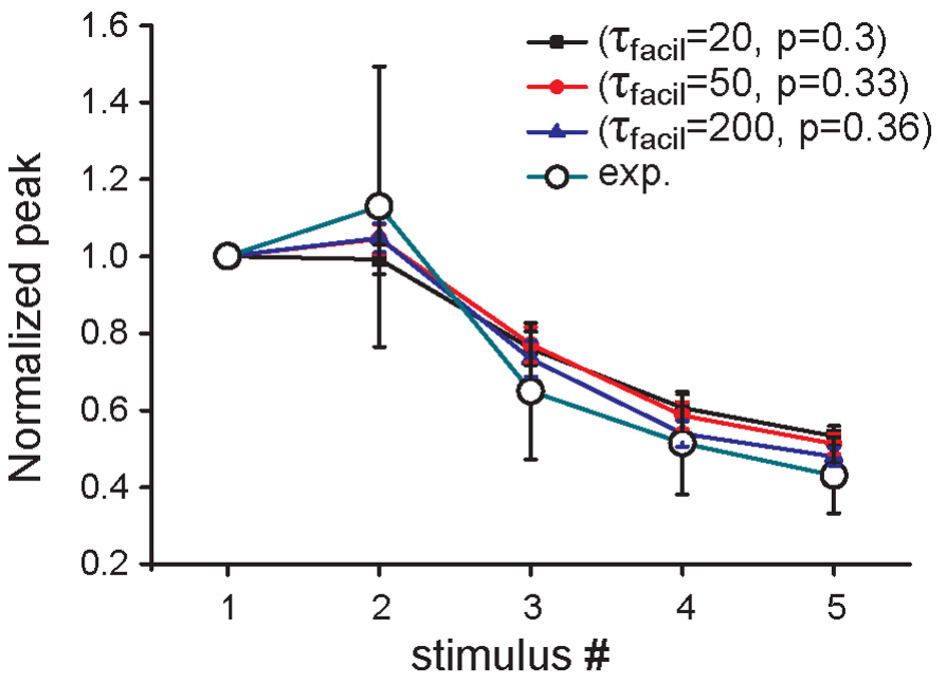
**Effect of increased Aβ on short-term plasticity.** Average relative somatic EPSC amplitude (soma clamped at −70 mV) against stimulus number, in the presence of an increased concentration of Aβ in experimental data (Abramov et al., 2009) (empty circles) and in three parameter configurations of the model, in which only *p*_0_ (blue curve) or both *p*_0_ and τ_facil_ are changed (black, red curves).

The same overall effect of Aβ on synaptic currents could in principle be obtained by assuming a different scenario in which, in addition to *p*_0_, at least another one of the parameters modulating the short-term synaptic dynamics is modified by Aβ accumulation. As an example, in Figure 3 we report results demonstrating how the same qualitative effect on synaptic responses is obtained by assuming that Aβ affects both the time constant for facilitation (τ_facil_) and *p*_0_, although by drastically different proportions. For instance, a 15% reduction in *p*_0_ (from 0.36 to 0.3) would require a corresponding 10-fold reduction in τ_facil_ (from 200 to 20) to reproduce the experimental findings. These results suggest that, even if a change in the release probability alone can explain the modulation of synaptic transmission by Aβ accumulation as suggested by experiments, additional mechanisms may also contribute to the overall effect.

### Predicting the effects of Aβ on short-term plasticity

**Figure 4:**
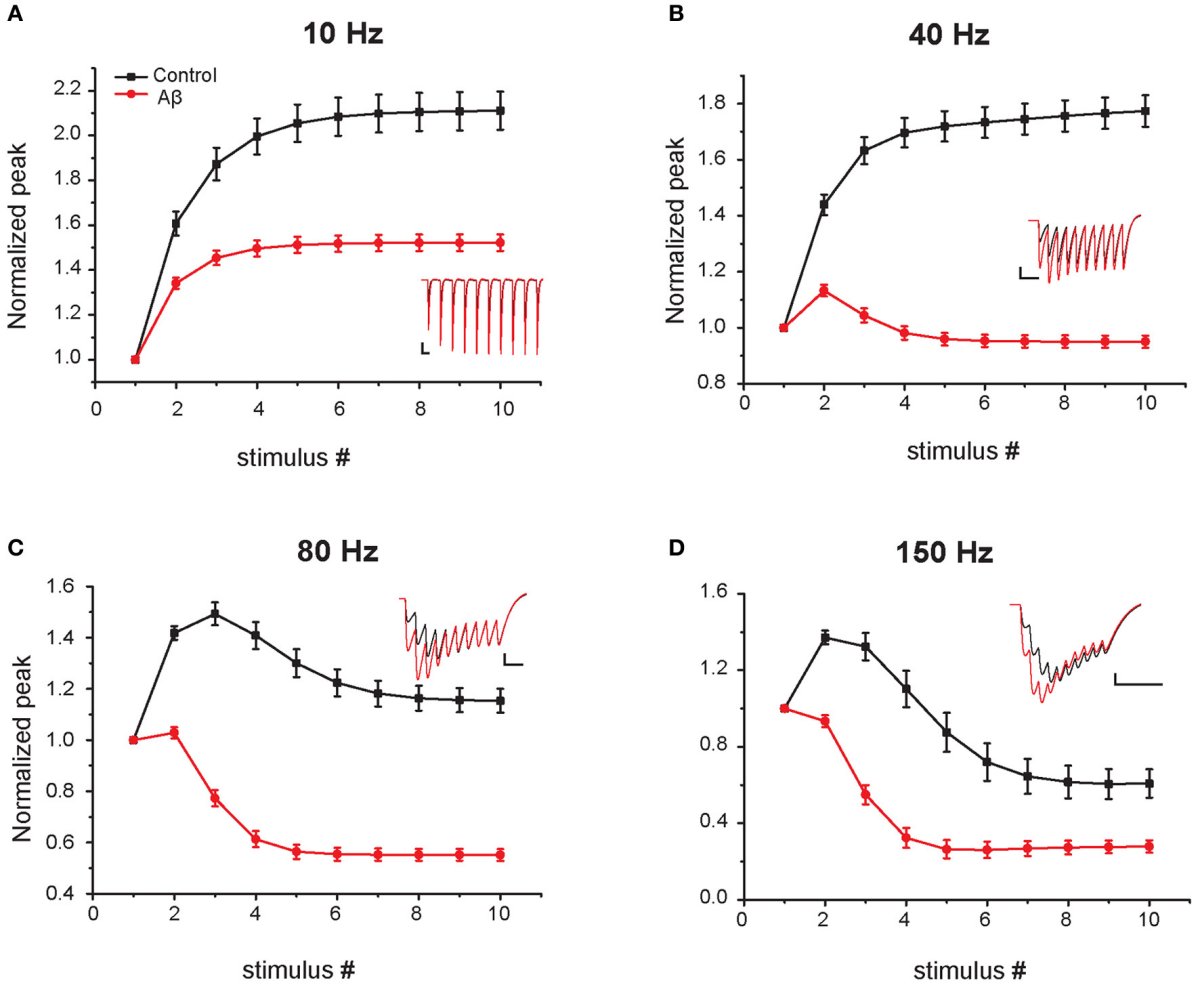
**Effect of Aβ on responses to repeated sub-threshold synaptic stimulation.** Average relative somatic EPSC amplitude in response to repetitive stimulation at different frequencies in the control (black) and increased-Aβ (red) model neuron (normalized to the first peak). The neuron was clamped at −70 mV and synapses stimulated 10 times at 5–200 Hz. Panels **(A–D)** show representative results obtained with an input frequency of 10 Hz **(A)**, 40 Hz **(B)**, 80 Hz **(C)**, and 150 Hz **(D)**. Relative somatic EPSC amplitude (soma clamped at −70 mV) was plotted against stimulus number. Synaptic weights were scaled in order not to evoke an action potential in the model post-synaptic neuron. Insets show example traces. Scale bars: 2 pA and 100 ms **(A)**, 50 ms **(B)**, 25 ms **(C–D)**.

In the experiments of Abramov et al. (2009), the effect of Aβ on short-term plasticity was evaluated during a train of five input stimuli at 100 Hz. To predict the effects of Aβ for a wider range of frequencies (from 5 to 200 Hz) and during a longer input train (10 synaptic stimuli), we carried out additional simulations using the same protocol used in experiments (i.e., somatic current recordings during voltage clamp at −70 mV). Figure 4 shows the average values over 10 trials (see “Materials and Methods”) for the relative EPSC amplitude in four representative cases. At 5–10 Hz both the increased-Aβ and control neuron show facilitation, a behavior that is more pronounced under control conditions. At 40 Hz the increased-Aβ neuron shows a bimodal behavior, with an initial facilitation followed by a depression after the 3–4th stimulus, while the control condition still displays only facilitation. At 80–100 Hz, both types of neurons display the bimodal behavior described above, with stronger depression in the increased-Aβ case. Finally, at 150–200 Hz, depression is favored over facilitation, with the latter totally absent in the increased-Aβ case. These results are in agreement with the notion that an enhancement in release probability will deplete synaptic vesicles more rapidly and thus favor synaptic depression over facilitation (Zucker and Regehr, 2002). The Aβ-induced alteration in release probability can therefore significantly alter the synaptic integration properties at different frequencies, and can be expected to interfere with the normal physiological and cognitive processes.

### Predicting the effects of Aβ-induced increase in release probability on CA1 neuronal output

**Figure 5:**
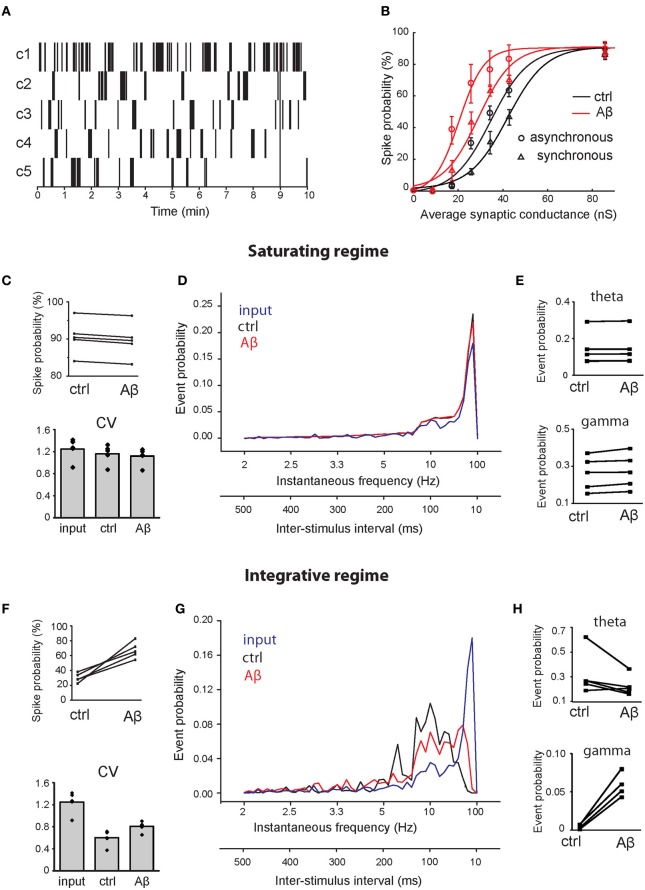
**Effect of Aβ on responses to repeated supra-threshold synaptic stimulation. (A)** Natural patterns from *in vivo* recordings of five CA3 pyramidal neurons used to stimulate synapses of the CA1 pyramidal neuron. **(B)** Spike probability (expressed as percentage) as a function of average peak synaptic conductance (nS), in control (black) and in the increased-Aβ neuron (red), when synapses are stimulated synchronously (circles) or asynchronously (triangles). The data sets were fitted with a sigmoid function *y* = *a*/[1 + *b* × exp(−*c* × *x*)] [black circle (control—synchronous inputs): *a* = 90, *b* = 64.96, *c* = 124.4; red circle (increased-Aβ—synchronous inputs): *a* = 90, *b* = 43.53, *c* = 188.4; black circle (control—asynchronous inputs): *a* = 90, *b* = 134.2, *c* = 0.1167; red triangle (increased-Aβ—asynchronous inputs): *a* = 90, *b* = 33.65, *c* = 0.1714]. Ten synapses were stimulated synchronously with the five natural patterns in two conditions: in a saturating regime (average peak synaptic conductance around 90 nS) **(C–E)** and in an integrative regime (average peak synaptic conductance around 30 nS) **(F–H)**. **(C,F)** Spike probability and CV of ISIs obtained with the five patterns. **(D,G)** Average instantaneous frequency distribution over the five patterns. An additional axis indicating the correspondent ISIs is included. **(E,H)** Spike probability to have an event in theta (4–8 Hz) and gamma (40–80 Hz) ranges.

CA1 pyramidal neurons receive the majority of their inputs from CA3 pyramidal neurons. To assess how the Aβ-induced increase in release probability influences CA1 pyramidal neuron output, we stimulated our control and increased-Aβ neuron with ‘natural’ input patterns obtained from *in vivo* recordings of rats during exploration of an open field. We used five different patterns of natural spike trains over a 10 min period (patterns c1–c5 in Figure 5A). A simulation used one pattern at a time, and each spike in the pattern activated 10 synapses randomly distributed on the apical trunk. We repeated this procedure 10 times for each pattern, randomly redistributing the 10 synapses and their weights. To evaluate the effect of Aβ on the neuron input-output relationship, we computed the average spike probability, defined as the ratio between the number of output spikes and the number of stimuli, and the interspike interval (ISI) distributions under control and increased-Aβ conditions.

Since the peak synaptic conductances (weights) during our experiments were not known, we tested the spike probability for a range of weights (from their default values to 10-fold higher), focusing on two possible scenarios: an ‘integrative’ and a “saturating” regime. In our model, we found that the relationship between spike probability and synaptic weights can be approximated by a sigmoid function in both the normal and the increased-Aβ conditions (Figure 5B—synchronous inputs). Figure 5B shows that when synaptic weights are low, the total synaptic current a neuron receives is never able to trigger an AP. As synaptic weights increase, the post-synaptic response is able to reach the threshold for AP generation when stimuli arrive grouped in bursts, i.e., the neuron needs to integrate several stimuli before reaching spike threshold (integrative regime). When synaptic weights are sufficiently high, almost every stimulus is able to generate a spike. In this condition, the neuron's capability to respond to pre-synaptic stimuli has reached saturation and a further increment in synaptic weights does not lead to an increment in firing probability (saturating regime). Note that the firing probability in this case does not reach 100% because many inputs fall within the refractory period and cannot trigger an AP. To distinguish the effect of Aβ under these different conditions, we studied two cases: (1) the integrative regime, corresponding to an average peak synaptic conductance of about 30 nS, where stimuli need to be summed to generate a spike, and (2) the saturating regime, corresponding to an average peak synaptic conductance of about 90 nS, where almost all stimuli generate a spike.

To test the effect of asynchronous inputs, we repeated the simulations for pattern #1 using a random activation of each synapse in the range 0–9 ms, corresponding to about one fourth of a gamma cycle (Figure 5B—asynchronous inputs). Asynchrony decreases spike probability in both the control and the increased-Aβ neuron, but the difference between the two cases remains qualitatively the same.

Under the saturating regime, the mean spike probability of the increased-Aβ neuron slightly but consistently decreased with respect to control (Figure 5C, ctrl = 90.55 ± 2.1%, Aβ = 89.63 ± 2.1%, paired *t*-test *p* < 0.001). The coefficient of variation (CV) of ISIs did not change significantly (Figure 5C, input = 1.25 ± 0.14, ctrl = 1.16 ± 0.12, Aβ = 1.12 ± 0.11, paired *t*-test *p* = 0.25). Furthermore, the instantaneous frequency distribution did not show any significant difference between the two conditions (Figure 5D, Kolmogorov–Smirnov test, *p* = 0.198). Within the theta (4–8 Hz) and gamma (40–80 Hz) ranges, there are small but significant differences (Figure 5E, paired *t*-test *p* = 0.04 in both cases). Under the integrative regime, which represents a more realistic biological environment, an increase in *p*_0_ caused an increase in the mean spike probability (Figure 5F, ctrl = 30.1 ± 2.7%, Aβ = 67.4 ± 10.8%, paired *t*-test *p* < 0.004). The CV of ISIs increased significantly (Figure 5F, input = 1.25 ± 0.14, ctrl = 0.60 ± 0.10, Aβ = 0.81 ± 0.07 paired *t*-test *p* = 0.005), due to an increase in higher frequencies, while lower frequencies remain unaltered (see below). In this case, the instantaneous frequency distribution was significantly different (Figure 5G, Kolmogorov–Smirnov test, *p* = 0.009), with Aβ enhancing firing frequencies in the gamma range (40–80 Hz) (paired *t*-test *p* = 0.001) and having no effect on firing frequencies in the theta range (4–8 Hz) (paired *t*-test *p* > 0.05) (Figure 5H). These results demonstrate that the increase in release probability induced by Aβ can significantly alter the response of a CA1 neuron to a natural stimulation pattern in a way that could influence the generation of behaviorally relevant brain rhythms.

## Discussion

An important integrative function for a neuron is to modulate its output firing rate to accurately represent the inputs it receives. Using a realistic model of CA1 pyramidal neurons, validated against experimental findings, we found that an increase in the synaptic release probability induced by Aβ accumulation can significantly change the firing patterns generated by a CA1 neuron. The Aβ-mediated increase in *p*_0_ reduces the range of stimulus strength over which the neuron can modulate its output in terms of firing rate, reaching the maximum firing probability much sooner than under control conditions. This change in synaptic transmission may be one of the early steps concurring to the overall degradation of cognitive functions mediated by hippocampal CA1 neurons in AD.

A number of other computational models have previously investigated the role of Aβ in AD. Previous reports investigated the Aβ-induced synapse and neuronal loss on hippocampal network dynamics and memory impairment (Ruppin and Reggia, 1995; Horn et al., 1996; Hasselmo, 1997; Rowan, 2012). We and others investigated the effects of Aβ on ionic channels regulating neuronal excitability and how these effects perturb hippocampal function (Morse et al., 2010; Zou et al., 2011; Culmone and Migliore, 2012). To our knowledge, the present report is the first computational investigation of how an acute Aβ-induced increase in presynaptic release influences neuronal output.

The findings of Abramov et al. showed an acute effect of Aβ (Abramov et al., 2009). The authors suggested that Aβ acts at the presynaptic terminal and increases the synaptic response by increasing the release probability. Other experimental models have evaluated the effect of Aβ on synaptic transmission with acute application of various forms of the Aβ peptide (synthetic, natural) and at different stages of the disease, i.e., in chronic conditions. Besides the acute effect on presynaptic release reported by Abramov et al. (2009), acute exposure to Aβ has also been shown to act at the postsynaptic terminal by reducing NMDA-mediated transmission (Snyder et al., 2005). In conditions where Aβ is chronically increased, such as in transgenic mouse models of the disease, both AMPA and NMDA glutamatergic transmission were either unaltered or reduced [reviewed in (Marchetti and Marie, 2011)]. It is likely that the effects of Aβ will be different at different stages of the disease and highly dependent on its aggregation status (e.g., monomers versus oligomers). It is conceivable that an acute increase in Aβ concentration could enhance glutamatergic transmission, which will have rapid effects on information processing as demonstrated by this study, but could also contribute to excitotoxicity, and epileptic phenomena. Its chronic accumulation, however, is likely to contribute to neuron degeneration and to a decrease in postsynaptic responses leading to reduced transmission by removal of AMPA and NMDA receptors from the synapses. It is becoming apparent from prior studies mentioned above and from this study that it will be essential to distinguish the physiological effects from the pathological effects of Aβ to effectively develop Aβ-based therapies to treat AD without disturbing normal cognition.

The results predicted by our model in response to natural stimuli are somewhat unexpected since the depressing effect on synaptic integration caused by Aβ (see Figure 2) would suggest a reduction in the spike probability during a train of synaptic inputs. The apparent contradiction can be explained by considering that, although the relative amplitude of the EPSC decreases during the train of synaptic stimuli, the absolute peak values of the EPSCs are higher under increased-Aβ conditions with respect to control (see Figure 4). This will generate more spikes at the beginning of the train under increased-Aβ conditions, i.e., for synaptic inputs depolarizing the membrane to values around the spike threshold, especially within an integrative regime.

Finally, the model predicts a different spike timing structure between control and increased-Aβ conditions in the theta and gamma range, during presentations of natural stimuli. As these frequencies have been associated with information processing, including memory formation (Colgin and Moser, 2010; Young, 2011), these results suggest that the Aβ-mediated changes in synaptic transmission can contribute to the altered cognitive functions observed during AD.

### Conflict of interest statement

The authors declare that the research was conducted in the absence of any commercial or financial relationships that could be construed as a potential conflict of interest.
